# Bureaucracy, happiness, and satisfaction at work

**DOI:** 10.1371/journal.pone.0338838

**Published:** 2026-01-22

**Authors:** Jeffrey Tu, Seth J. Hill

**Affiliations:** 1 University of California San Diego, La Jolla, California, United States of America; 2 Department of Political Science, University of California San Diego, La Jolla, California, United States of America; Universiti Putra Malaysia, MALAYSIA

## Abstract

Despite increasing material prosperity, industrialized nations face declining self-reported happiness and increasing workplace dissatisfaction. This study investigates bureaucratic burden as a driver of diminishing job satisfaction, analyzing 7.9 million Glassdoor reviews of more than 8,000 companies from 2008-2023 using natural language processing and instrumental variables methods. We identify reviews mentioning bureaucracy and quantify their association with 1-5 star employer ratings. Mentioning bureaucracy corresponds to around 0.7-point lower ratings (a 22% decline from the mean), comparable to the impacts of mentioning low pay (–0.8) or workplace conflict (–0.9). Two-stage least squares analysis, instrumenting with future bureaucratic mentions at the same company, implies a causal relationship. These findings support theories about the harm of “illegitimate tasks” at work and suggest revisiting conventional efficiency rationales for workplace bureaucratization. Organizational practices emphasizing employee autonomy and meaningful tasks could partly mitigate declines in satisfaction.

## Introduction

**Fig 1:**
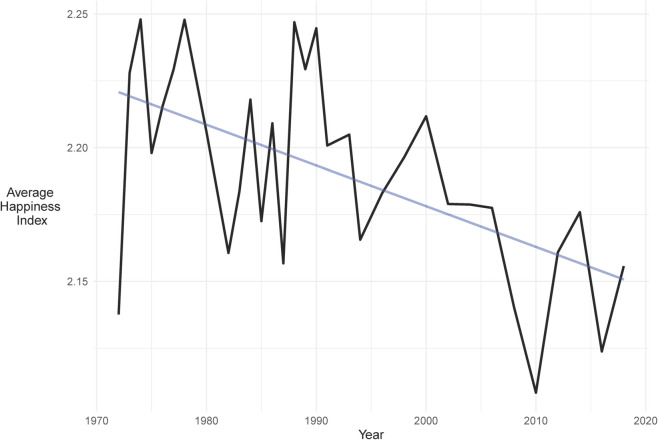
Subjective happiness in the United States.

Individuals across the developed world report increasing levels of unhappiness. Fig 1, for example, presents a decline in reported happiness by American respondents to the General Social Survey [1] from 1972 through 2023. What caused the decline in life satisfaction? The decline is unlikely to have been caused by changes in income or consumption. Consumption per capita in the United States increased by almost 200% during this time period [2] and evidence suggests that increasing income corresponds to greater subjective well-being at all income levels [3].

A recent review of evidence from psychology found that sense of gratitude and extent of social relationships are the most common factors of individual happiness [4]. Other research finds that exchanging wealth for free time as well as more time in nature increase happiness [5,6], suggesting that the time pressures of modern life could be one factor in declining satisfaction [7].

A second source of dissatisfaction is experience at work. Most adults spend a third of their waking hours working. Research shows that dissatisfaction in the workplace spills over into dissatisfaction outside of work and that meaningful work through “earned success” is an essential ingredient to human happiness [8–10]. In recent years, many adults report their jobs feel increasingly meaningless [11] and the COVID-19 pandemic and its aftermath saw record turnover of employment in the United States, the so-called Great Resignation [12].

Previous research suggests that multiple factors affect well-being at work, including a sense of community, value alignment, workload management, and a feeling of accomplishment [13,14]. Hybrid work, where employees spend part of their work week from home, improves job satisfaction and decreases quit rates [15]. Recent evidence points to “illegitimate tasks”—burdens that workers view as unnecessary or peripheral to their core mission—as a particular detriment to satisfaction. Peripheral administrative work correlates with increased burnout, lower engagement, and reduced life satisfaction [16–18]. Illegitimate tasks might be even more harmful than long hours as physicians identified mounting paperwork as larger sources of exhaustion than long hours during the COVID-19 pandemic [19,20].

In this paper, we investigate the relationship between bureaucracy at work and job satisfaction. We imagine bureaucracy as the time spent on paperwork or training peripheral to the employee’s actual role and/or layers of administration that impede autonomy and decision-making. The company itself need not be structured as a bureaucracy for employees to feel burdened by administrative tasks or bureaucratic hurdles.

On one hand, standardization of recurring employee tasks, i.e., bureaucracy, could improve organizational efficiency, reduce worker stress, and increase satisfaction at work. On the other hand, excessive administrative burden could impede workers’ sense of autonomy and accomplishment thereby preventing them from achieving “earned success” and finding meaning in work. This tension is particularly acute for knowledge workers, whose productivity depends upon creativity, self-esteem, and independent judgment.

An investigation more than four decades ago [21] found that increased bureaucratization led to lower satisfaction with work for public employees in Virginia. Their conclusions, however, were criticized for unstable correlations and potential confounding factors [22]. Standardization and ambiguity harmed satisfaction while autonomy improved satisfaction in an analysis of survey responses from 169 administrators in six states in 1984-85 [23].

Here, we update and expand these findings using 7.9 million Glassdoor reviews of places of work from 2008 to 2023 to examine how bureaucratic burden affects worker satisfaction. These reviews include open-entry text written by each reviewer summarizing their experience with the company. We test whether reviewers who mention bureaucracy or bureaucratic burdens in their reviews report lower overall satisfaction scores compared to reviewers who do not. This relationship would support theories connecting meaningful work to earned success and illegitimate tasks to dissatisfaction.

We find that reviews that mention bureaucracy give overall ratings 20% lower than reviews that do not. To benchmark this magnitude of dissatisfaction, we compare the relationship between mentioning bureaucracy and company rating to the relationship with mentioning long hours, low pay, conflict, and stress. We find that bureaucracy has a comparable negative relationship to satisfaction as each of these benchmarks, with only conflict consistently corresponding to lower ratings than bureaucracy. These comparisons, in one way, are conservative, as conflict or stress could be *caused* by the burdens of bureaucracy in an organization.

To address concerns about reverse causality—where those who dislike an employer rationalize their distaste by mentioning every negative thing they imagine might make a workplace frustrating, such as bureaucracy—we present a two-stage least-squares instrumental variables analysis (2SLS). We instrument for mentioning bureaucracy in a review by the average rate other reviewers of the same company mention bureaucracy in the 365 days that follow the review. Reviewers cannot see other reviews until they submit their own (what Glassdoor calls a *give-to-get* approach). The 2SLS analysis supports a causal relationship between experience with bureaucracy at work and dissatisfaction.

Our results suggest that experience with bureaucracy is an important factor in dissatisfaction at work. If meaningful experiences at work are an important input to overall life satisfaction, and if the transition to a service economy led to increasing experiences of bureaucracy at work over the last 50 years, bureaucracy could be part of the explanation for the decreased satisfaction in today’s industrialized economies.

## Results

**Fig 2:**
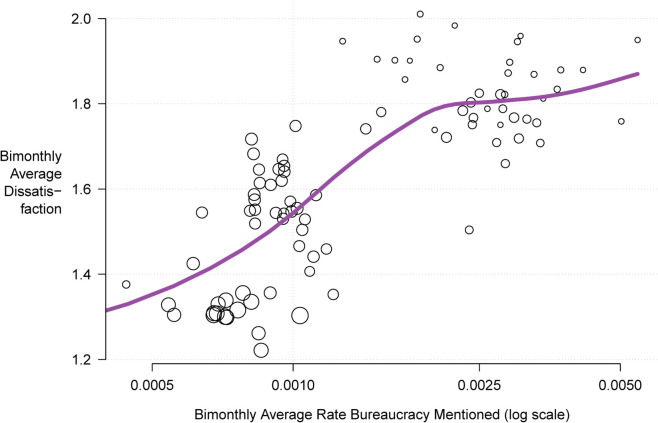
Glassdoor reviewer mentions of bureaucracy and company ratings, 2008–2023.

In Fig 2, we present a scatter plot of our key outcome and explanatory variables. The x-axis presents the rate (logarithmic scale) that Glassdoor reviewers mention bureaucracy in the text summary of the company. To construct this measure, we ran a search on each text summary for the string ‘bureau’. The rate is the proportion of headline summaries with a match for that string search. The y-axis represents average dissatisfaction coded as five minus each reviewer’s five-star overall rating of the company. Each point plots the two variables aggregated to two-month periods with point size proportional to the number of reviews in that period. Overlaid above the points is a loess smooth best-fit line.

Fig 2 shows a positive relationship between bureaucracy and dissatisfaction at the aggregate level. Averaging across all companies and reviews, increasing monthly averages of bureaucracy mentions correspond to increasing levels of company dissatisfaction.

**Fig 3:**
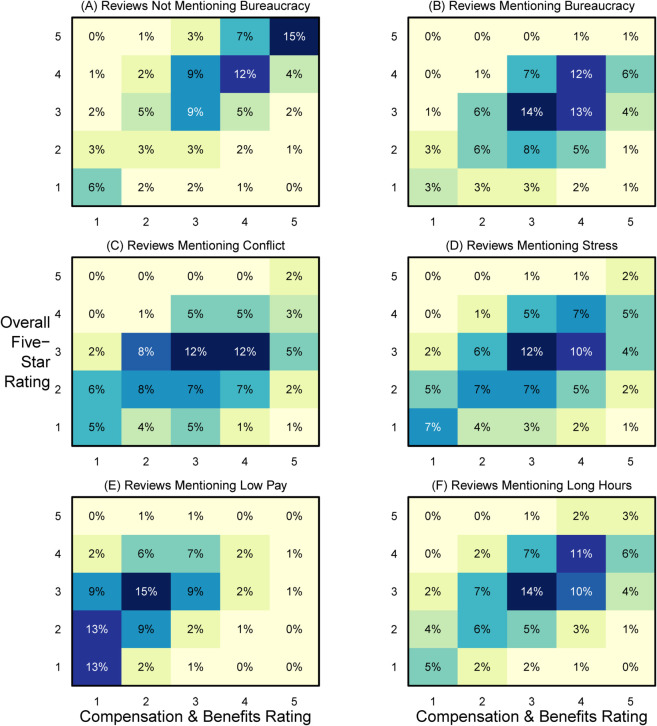
Joint distribution of overall rating for reviews mentioning comparison factors. Note: Cell shading corresponds to joint density, cell percentages of the table.

In Fig 3 we evaluate the relationship between bureaucracy and company rating at the individual level. We present joint distributions of five-star rating of the company and the five-star rating of the company’s compensation and benefits as heatmaps. The top left facet presents the distribution for the reviews in the data set that do not mention bureaucracy in the headline of their review. The top right facet presents the distribution for the reviews that mention bureaucracy in their headline. Cells are shaded corresponding to the joint density of reviews with cell text presenting the percentage of reviews at that cell.

Comparing patterns in the two facets in the top row of Fig 3 highlights how experience with bureaucracy harms satisfaction at work. While the modal overall rating for those not mentioning bureaucracy in their review is five stars (26% of reviews), the modal rating for those mentioning bureaucracy is three stars, with only 2% giving five stars. An additional 28% of reviews not mentioning bureaucracy give four stars compared to 26% of reviews that do.

To benchmark the magnitude of the relationship between bureaucracy and satisfaction, the four facets in the second two rows of Fig 3 present joint distributions for reviews that mentioned the strings ‘conflict’ (middle left facet), ‘stress’ (middle right), ‘low pay’ (bottom left), or ‘long hours’ (bottom right). Reviews mentioning these negatives each have modal reviews, like those mentioning bureaucracy, of three stars. Two percent of reviews mentioning conflict and low pay, respectively, give five-star ratings to the company, while 4% and 6% of reviews mentioning stress and long hours give five stars. Comparison across the heatmaps of Fig 3 suggests that bureaucracy is of similar harm to satisfaction as conflict and low pay but somewhat less harmful than stress or long hours.

**Fig 4:**
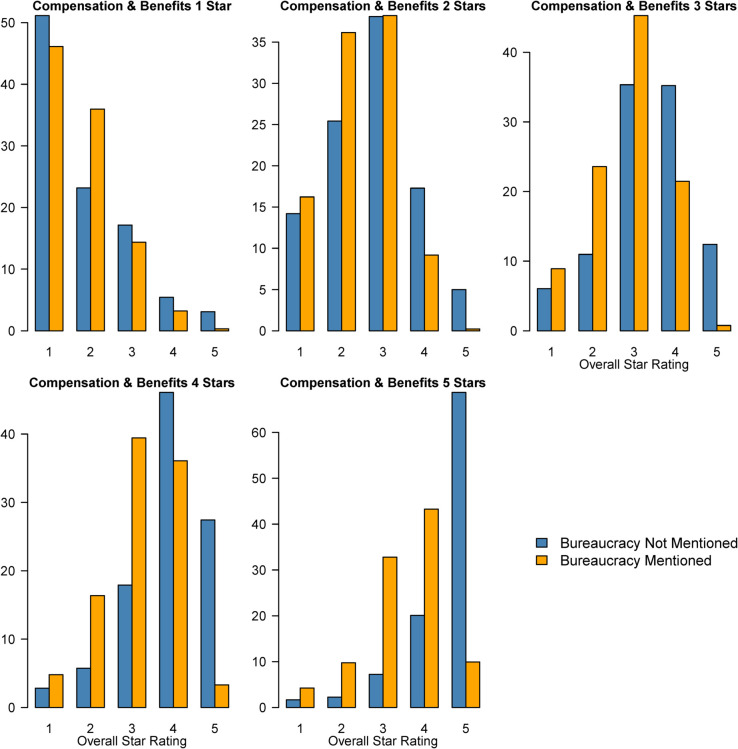
Distribution of overall company rating by rating of compensation and benefits and by mention of bureaucracy.

To directly compare reviews that mention bureaucracy to those that do not, in Fig 4 we present bar plots of overall rating (x-axis) by rating of compensation and benefits from one to five stars (each facet) separately for reviews that do and do not mention bureaucracy (bar pairs). For example, the facet in the top right shows that, among reviewers who rated the company’s compensation and benefits with three stars, around 15% of those who did not mention bureaucracy gave the company a five-star overall rating (left bar above the number five) compared to around 1% of those who did mention bureaucracy (right bar above the number five). The two bars above the number four indicate that about 35% of those who did not mention bureaucracy versus about 20% of those who did mention bureaucracy gave a four-star review.

At each rating of compensation and benefits, a much larger proportion of reviews that did not mention bureaucracy give five-star ratings than do reviews that did mention bureaucracy. Only when compensation and benefits receive five stars do reviews mentioning bureaucracy give four stars at a higher rate than reviews that do not.

In total, Figs 3 and 4 show that experience with bureaucracy leads to lower satisfaction with the company at magnitudes similar to experiences with conflict, stress, and low pay as well as at all levels of compensation and benefits.

### Regression analysis

Figs 3 and 4 show that mentioning bureaucracy in the summary review corresponds to significantly lower overall ratings of the company and does so at a magnitude similar to that of mentioning conflict, stress, long hours, and low pay. To estimate the marginal contribution of each factor, we turn to least-squares regression. In Table 1, we present regression estimates of the relationship between mentioning bureaucracy and the rating given to the company. The outcome variable is the five-star rating of the company by the reviewer. The first column presents the bivariate relationship only, the second adds a control for compensation and benefits, the third adds controls for other text strings in the review, and the fourth and fifth columns include company and year fixed effects. These fixed effects hold constant fixed features of firms and average changes across years.

**Table 1:** Bureaucracy mentions and overall rating of employer.

	(1)	(2)	(3)	(4) Company + Year FE	(5) Company + Year FE
Bureaucracy mentioned in headline	−0.649*** (0.014)	−0.594*** (0.011)	−0.648*** (0.014)	−0.570*** (0.010)	−0.735*** (0.013)
Numeric rating of compensation and benefits		0.634*** (0.000)		0.630*** (0.000)	
Low pay mentioned in headline			−0.832*** (0.009)		−0.746*** (0.008)
Long hours mentioned in headline			−0.465*** (0.012)		−0.396*** (0.012)
Conflict mentioned in headline			−0.939*** (0.051)		−0.807*** (0.048)
Stress mentioned in headline			−0.806*** (0.008)		−0.663*** (0.007)
Constant	3.519*** (0.000)	1.387*** (0.001)	3.524*** (0.000)	1.398*** (0.001)	3.524*** (0.000)
Observations	7,902,868	7,902,868	7,902,868	7,902,868	7,902,868
R-squared	0.000	0.397	0.003	0.440	0.101
Adjusted R-squared	0.000	0.397	0.003	0.439	0.100
F-statistic	2189.11	2599253.40	5007.47	2400403.58	4318.23

Robust standard errors in parentheses.

* *p* < 0.10, ** *p* < 0.05, *** *p* < 0.01.

Across specifications, we find reviews that include the text string ‘bureau’ have overall ratings of the company about 0.6 or 0.7 points lower on the five-star scale than reviews that do not have that string. With mean ratings of about 3.3, this implies a proportional decline in overall rating of almost 20%. The point estimate attenuates when we control for the reviewer’s star rating of Compensation and Benefits but increases in magnitude when we include company and year fixed effects.

Columns three and five allow us to benchmark the relationship between bureaucracy and rating to the relationships between other text strings and rating. In column three, we find that bureaucracy is about 30 percent less harmful to satisfaction than conflict, stress, and low pay but about 40 percent more harmful than long hours. Holding constant features of each company (column five), we find that bureaucracy is again less harmful than conflict but now about equally harmful as low pay and stress.

In S3 Table, we estimate the specifications of Table 1 columns five and six subset by five-year time periods. The negative effect of bureaucracy appears to be increasing over this time period, with the coefficients for 2019 to 2023 about 50% larger than for 2008 to 2012.

### Instrumental variables results

One concern with analysis to this point is reverse causality. Our theory is that the level of bureaucracy in the company causes a less meaningful job, which causes a lower rating by the reviewer of the company. An alternative interpretation of our evidence, however, is reverse causality where the reviewer’s dissatisfaction with the company causes them to come up with justifications for their low rating such as bureaucracy (or other negative features of firms) to write in their review.

To address concern about reverse causality, we use a two-stage least-squares (2SLS) instrumental variables approach. To instrument for the level of bureaucracy experienced by the reviewer in their company, we use the rate that bureaucracy is mentioned in Glassdoor reviews of that same company in the 365 days following the reviewer’s (see discussion in the Materials and Methods section). In Table 2, we present results from the 2SLS analysis. Column one presents the first stage estimates, revealing that the 365-day forward average rate that subsequent reviewers mention bureaucracy strongly predicts the endogenous variable. The F-statistic is 46. Column two presents the 2SLS estimate, suggesting that experiencing bureaucracy at a firm such that the reviewer mentions it in the headline of their review causes more dissatisfaction with the company. The results in Table 2 reinforce a causal interpretation of bureaucracy on satisfaction at work.

**Table 2:** Instrumental variable results: Bureaucracy mentions and overall rating of employer.

	(1) First Stage	(2) IV: Rating
Bureaucracy mentioned in headline		−7.182** (3.101)
Forward average bureaucracy mentioned in firm reviews	0.182*** (0.017)	
Numeric rating of compensation and benefits	−0.000*** (0.000)	0.625*** (0.004)
Total reviews of firm (log)	0.000 (0.000)	0.032*** (0.008)
Constant	0.001*** (0.000)	1.152*** (0.052)
Observations	6,764,031	6,764,031
R-squared	0.001	0.360
Adjusted R-squared	0.001	0.360
F-statistic	46.33	

Robust standard errors clustered on firm in parentheses.

* *p*< 0.10, ** *p*< 0.05, *** *p*< 0.01.

## Discussion

Our results indicate that experiencing bureaucracy at work causes employees to evaluate their employer less favorably. The significance of this relationship is comparable to the effects of more established components of job satisfaction such as long hours, stress, conflict, and low pay.

A recent book [8] argues that two intrinsic goals lead to earned success at work. First, improving one’s skill and effectiveness at work. Second, a belief that one’s work serves the needs of others. Bureaucratic tasks both limit the ability to improve skills and take time away from serving others.

Our large Glassdoor dataset overcomes the small sample sizes and single-industry focus of previous work comparing experience with bureaucracy and job satisfaction. We also cover a longer time period and move beyond correlational evidence by using 2SLS analysis.

Our findings have some limitations. First, the study relies on self-reported perceptions of bureaucracy at work. Objective measures of workplace bureaucracy would complement the analysis here but are difficult to construct. This seems a promising opportunity for future research. Even so, individual perception of bureaucratic burden might be more relevant than objective bureaucratic burden with respect to employee satisfaction. Second, although we analyze 7.9 million observations covering most large industries, Glassdoor Economic Research reports that the Information industry is significantly overrepresented in its data while Public Administration and Wholesale Trade are underrepresented [24]. It is possible that Public Administration could merit particular attention.

Our results point to several areas of further research in organizational studies and happiness research. Studies should continue to analyze the relationship between more specific facets of bureaucracy and happiness. We use a somewhat rough indicator of bureaucracy from the Glassdoor text. More nuanced metrics of bureaucracy would analyze the relative influence of different aspects of bureaucracy on earned success and satisfaction. For example, one might use a bureaucracy scale which includes “division of labor, hierarchy of authority, impersonality, the existence of definite procedures concerning work, and the degree to which technical competence serves as a basis for hiring and promotion” [21]. Further studies could use more complex text analysis to identify reviews that demonstrate these factors and analyze their relation to satisfaction.

These results support theories that frame experience with bureaucracy as “illegitimate tasks,” which undermine employees’ sense of autonomy and meaningful contribution. Given the broader trends of declining happiness in industrialized societies, our study suggests that addressing excessive bureaucracy could play a role in improving workplace satisfaction and, by extension, overall well-being. Future research should further investigate the specific dimensions of bureaucratic burden that most erode job satisfaction and explore organizational strategies that balance necessary administrative structure with employees’ need for autonomy and purpose.

## Materials and methods

### Data collection and processing

We use a database of company reviews from the employment website Glassdoor with approximately 7.9 million reviews in the years 2008 to 2023 [25]. This dataset includes job-related details, 5-star overall rating of the company, categorical ratings of other aspects of the company, and text from three open-entry text fields of headline summary, pros, and cons. Our analysis focuses on the headline text because we imagine this to represent the most salient features of the reviewer’s experience.

Glassdoor Economic Research discusses potential sources of bias in the data [24]. Glassdoor accounts for selection bias with a give-to-get model where users are required to submit reviews prior to accessing other users’ content, which has been shown to improve the representativeness of the reviews [26].

### Outcome variable

Our outcome variable is the overall rating of the company, measured by the reviewer’s selection from 1 to 5 stars. The highest and lowest rated companies with at least 100 reviews in the data set are Radio Flyer and Helios & Matheson IT, with average ratings of 4.9 and 1.8 stars, respectively.

### Explanatory variables

We used string matching on the text fields of each Glassdoor headline text to measure our explanatory variables. We searched for the string ‘bureau’ to measure if the reviewer mentioned bureaucracy or bureaucratic burden in their review. We also searched for the strings ‘low pay,’ ‘long hours,’ ‘conflict,’ and ‘stress.’ We use as a control variable the reviewer’s rating of ‘Compensation & Benefits’ from 1 to 5 stars. In some specifications we include company and year fixed effects to hold constant fixed features of each company and calendar year.

S1 Table presents summary statistics for outcome and explanatory variables. S2 Table presents text from a random sample of ten reviews that match our string search for ‘bureau.’ It is clear that most reviews that mention bureaucracy in their headline summary do so with negative connotation.

### Statistical approach

Our main regression specification is

$$Rating_{ict}=\;\alpha_{c}+\delta_{t}+\beta \;MentionsBureaucracy_{i}+\gamma_{1}\;CompBenefits_{i}\;+\gamma_{2}\;OtherStrings_{i}+\varepsilon_{i}$$ (1)

where *i* indexes a rating, *c* indexes the more than 8,000 or so companies covered by our data set, *t* indexes calendar year, *Rating*_*ict*_ is review *i*’s 5-star-scale rating of company *c* posted in year *t*, *α* is a company fixed effect, *δ* is a year fixed effect, *MentionsBureacracy*_*i*_ is a 0/1 indicator variable taking the value of 1 when the review mentions bureaucracy in the text, *CompBenefits*_*i*_ is the review’s 5-star rating of the company’s compensation and benefits, *OtherStrings*_*i*_ are separate 0/1 indicators for the presence of each of the other strings mentioned in the previous paragraph, *β* and *γ* are regression coefficients, and *ε* is an idiosyncratic error term. In all estimations, we cluster standard errors on the company.

### Reverse causality and instrumental variables

Our instrument for the level of bureaucracy experienced by the reviewer in their company is the rate that bureaucracy is mentioned in Glassdoor reviews of that same company in the 365 days following the review. This instrument meets the two criteria for an instrumental variable. First, the instrument is correlated with the endogenous variable because the future rate that bureaucracy is mentioned in reviews should be correlated with the reviewer’s own experience of bureaucracy in the firm. Second, the exclusion restriction holds because, on the assumption that time moves in only one direction, future reviews cannot cause the reviewer to mention bureaucracy. Glassdoor’s requirement that users post their own review before accessing others further supports the exclusion restriction.

The first-stage of the 2SLS specification is

$$MentionsBureaucracy_{itc}=\;\eta_{c}+\tau_{t}+\lambda_{1}\;MentionsBureaucracy_{c,t+1,t+366}\;+\lambda_{2}\;CompBenefits_{i}+\lambda_{3}\;log(NumberReviews_{c})\;+\nu_{itc}$$ (2)

where *t* indexes calendar date, *MentionsBureaucracy*_*c*,*t* + 1,*t* + 366_ measures the rate that reviewers of company *c* on calendar dates from one to 366 days after the current review mention bureaucracy, $NumberReviews_{c}$ counts the number of total reviews for company *c* to account for differential measurement error, *η* and *τ* are company and year fixed effects, *λ* are regression coefficients, and ν is an idiosyncratic error term.

The second-stage equation of the 2SLS estimates

$$Rating_{itc}=\;\alpha_{c}+\delta_{t}+\beta_{2}\;{\hat{MentionsBureaucracy}}_{tc}+\gamma_{1}\;CompBenefits_{i}\;+\gamma_{3}log(NumberReviews_{c})+\varepsilon_{it}$$ (3)

where ${\hat{MentionsBureaucracy}}_{tc}$ is the predicted value from the first stage. Under the assumptions of 2SLS, $\beta_{2}$ returns the effect of mentioning bureaucracy purged of reverse causality. Standard errors are again clustered on company.

## Supporting information

S1 File Bureaucracy_and_Happiness_SI.pdf. Summary statistics for each of the variables included in the final analysis; Example headline text mentioning bureaucracy; Bureaucratic dissatisfaction over time.
(PDF)
