# Supplementary material for: Bureaucracy, happiness, and satisfaction at work
Source: PLoS One. 2026 Jan 22;21(1):e0338838. doi: 10.1371/journal.pone.0338838 (PMC12826465; doi:10.1371/journal.pone.0338838)
Supplement: S1 File — Summary statistics for each of the variables included in the final analysis; Example headline text mentioning bureaucracy; Bureaucratic dissatisfaction over time. (PDF) [file pone.0338838.s001.pdf]

Supporting information:
"Bureaucracy, happiness, and satisfaction at work"
PLOS One

Jeffrey Tu[1], Seth J. Hill[2*]

1 Class of 2026, University of California San Diego, La Jolla, California, United States
2 Professor, Department of Political Science, University of California San Diego, La Jolla, California, United States

* sjhill@ucsd.edu

Supporting information

S1 Table. Summary statistics for each of the variables included in the final analysis. The 'bureau' string was detected in about 1.2% of reviews.

Mean	SD	Variable
0.01	0.11	Bureaucracy mentioned in headline
3.36	1.26	Numeric rating of compensation and benefits
0.03	0.16	Long hours mentioned in headline
0.02	0.14	Low pay mentioned in headline
0.002	0.05	Conflict mentioned in headline
0.03	0.17	Stress mentioned in headline
3.56	1.25	Overall Rating of Employer

S2 Table. Example headline text mentioning bureaucracy.

Firm	Text
Scotiabank	good firm, lots of bureaucracy
State of Alaska	lots of bureaucracy
Deloitte	stimulating at times, but face-time and bureaucracy an issue
William Hill	good experience but very bureaucratic
Freddie Mac	great learning experience...strong culture with years of bureaucracy
US Air Force	great opportunities, obnoxious bureaucracy
Massachusetts General Hospital	good teammates but pathetic middle managers and bureaucratic behaviors
TSA Transportation Security Administration	government bureaucracy
National University of Singapore	terrible bureaucracy, incompetent secretaries, very variable quality of faculty
Deutsche Bank	bureaucracy, meritocracy and capitalism

S3 Table. Bureaucratic dissatisfaction over time. The relationship between bureaucracy and satisfaction at work over time. We separately estimate the specifications of columns five and six of Table 1 for years 2008 to 2012 (columns one and two of Table 1), 2013 to 2018 (three and four), and 2019 to 2023 (five and six). The negative effect of bureaucracy appears to be increasing over this time period, with the coefficients for 2019 to 2023 about 50% larger than for 2008 to 2012.

	2008 to 2012		2013 to 2018		2019 to 2023	
	(1)	(2)	(3)	(4)	(5)	(6)
Bureaucracy mentioned in headline	-0.473***	-0.525***	-0.509***	-0.642***	-0.662***	-0.900***
	(0.034)	(0.039)	(0.017)	(0.019)	(0.021)	(0.023)
Numeric rating of compensation and benefits	0.619***		0.615***		0.634***	
	(0.005)		(0.006)		(0.005)	
Low pay mentioned in headline		-0.511***		-0.628***		-0.820***
		(0.038)		(0.017)		(0.014)
Long hours mentioned in headline		-0.172***		-0.314***		-0.474***
		(0.041)		(0.020)		(0.018)
Conflict mentioned in headline		-0.768***		-0.690***		-0.912***
		(0.171)		(0.060)		(0.076)
Stress mentioned in headline		-0.434***		-0.575***		-0.753***
		(0.029)		(0.018)		(0.015)
Constant	1.204***	3.221***	1.363***	3.357***	1.478***	3.685***
	(0.015)	(0.000)	(0.019)	(0.000)	(0.017)	(0.000)
Observations	326,949	326,949	3,084,542	3,084,542	3,882,406	3,882,406
R-squared	0.412	0.146	0.418	0.107	0.450	0.090
Adjusted R-squared	0.401	0.130	0.416	0.104	0.449	0.088
F-statistic	8993.66	123.65	6073.32	563.52	9733.73	1227.39
Mean of Outcome	3.21	3.21	3.35	3.35	3.68	3.68
Company FEs	Yes	Yes	Yes	Yes	Yes	Yes
Year FEs	Yes	Yes	Yes	Yes	Yes	Yes

Robust standard errors clustered on firm in parentheses
* $p < 0.10$, ** $p < 0.05$, *** $p < 0.01$
